# Intravenous immunoglobulin (IVIG) therapy in pregnancies complicated by acute Parvovirus B19 infection in the second trimester: a case series

**DOI:** 10.1515/crpm-2025-0010

**Published:** 2025-09-11

**Authors:** Rachel Lee, David Garry, Kimberly Herrera, Jennifer Choi, Cassandra Heiselman, James Bernasko, Zenobia Gonsalves, Emily Stetler, Tiffany Yang, Elizabeth Garduno, Cecilia Avila

**Affiliations:** Department of Maternal Fetal Medicine, Stony Brook University Hospital, Stony Brook, NY, USA; Department of Obstetrics and Gynecology, Stony Brook University Hospital, Stony Brook, NY, USA

**Keywords:** acute Parvovirus B19, IVIG therapy, MCA-PSV, reticulocyte count, second trimester of pregnancy

## Abstract

**Objectives:**

Intravenous immunoglobulin (IVIG) in pregnancy has been used to treat hematologic conditions, but there is limited literature on its use in acute Parvovirus B19 infection. The purpose of this report is to highlight IVIG treatment recommendations based on low reticulocyte counts and high middle cerebral artery peak systolic velocity (MCA-PSV) values to prevent fetal hydrops, as well as identify which patients would benefit from high-dose IVIG administration in the setting of acute Parvovirus B19 infection in the second trimester.

**Case presentation:**

We present the treatment of acute Parvovirus B19 infection with IVIG in two affected pregnancies, as an alternative to percutaneous umbilical blood sampling (PUBS) and intrauterine transfusion (IUT).

**Conclusions:**

IVIG may be useful for Parvovirus B19 treatment in patients with erythropoietic suppression, in patients who decline PUBS/IUT with elevated MCA-PSV, and in patients who have difficult access to the placental cord insertion site, unsuccessful attempt at PUBS, or relative contraindications (i.e. significant abdominal surgery, high BMI).

## Introduction

Acute Parvovirus B19 infection in pregnancy can result in miscarriage, stillbirth, severe fetal anemia, non-immune fetal hydrops, and transient maternal bone marrow suppression [1], 2]. Parvovirus B19 infection in pregnancy is managed by ultrasound surveillance and treatment of severe fetal anemia with IUT. Currently, IVIG use for Parvovirus B19 is limited to case reports. The definitive mechanism of IVIG in the setting of Parvovirus B19 is unclear, but a possible explanation is that IVIG contains neutralizing antibodies that reduce viral load and modifies the hematopoietic response [3]. While IVIG readily crosses the placenta, safety of its use has been well documented [4].

On August 13, 2024, the Centers for Disease Control and Prevention (CDC) issued a health advisory to notify healthcare providers about a recent increase in Parvovirus B19 activity in the United States, and the Society for Maternal-Fetal Medicine (SMFM) issued an update on August 27, 2024, signifying an urgent need for treatment modalities given a rise in incidence [5]. IVIG may be a superior treatment option given its associated antiviral properties in reducing viral replication and modifying the hematopoietic response without the risks associated with invasive fetal procedures.

In this case series, we present the treatment of acute Parvovirus B19 infection with IVIG in two affected pregnancies to reduce viral load and prevent bone marrow suppression.

## Case presentation

### Case 1

#### Symptoms/signs

35-year-old G4P2012 with prior uncomplicated pregnancies presented to the hospital at 21 weeks’ gestation with 5 days of fever, chills, diaphoresis, headache, nausea, and weight loss of 4 lbs. in the span of 1 week. Past medical history was significant for alpha thalassemia silent carrier, band-3 deficiency spherocytosis, and history of hemolytic anemia in the setting of prior cytomegalovirus (CMV) infection (with previous negative IgG serologies for Parvovirus).

#### Diagnosis

Laboratory analysis demonstrated pancytopenia and hemolytic anemia. Evaluation was consistent with acute Parvovirus B19 infection with positive PCR and serologies (IgM 21.07, IgG 0.61), elevated from serology 5 years prior (IgM 0.23, IgG 0.37). Her reticulocyte count (baseline 8–10 %) decreased to 0.74 %, which was consistent with acute Parvovirus B19-induced hematopoietic suppression. Amniocentesis and MCA-PSV were deferred given acuity of the situation.

#### Treatment

Patient received high dose IVIG 1 g/kg daily for 3 doses (total 270 g). She also was transfused 3 units packed RBCs secondary to her low hemoglobin/hematocrit (H/H) of 6.7/17.9 and low reticulocyte count <10 %.

She received the first IVIG dose 7 days from the onset of symptoms. Admission viral load was >7 log IU/mL. After IVIG, we noted recovery of bone marrow suppression as evidenced by improving cell line recovery: WBC count rose from 2.42 to 18.15; platelets from 115 to 435 (4 days post-IVIG). Appropriate rise in H/H was noted post-blood transfusion. There was no change in percentage of immature granulocytes during this time, however 3 months post-IVIG the reticulocyte count improved to the patient’s baseline (9.13 %).

#### Follow-up

Weekly sonograms including MCA-PSV were performed to assess for fetal anemia and fetal hydrops for 12 weeks. MCA-PSV remained normal without a need for IUT.

The patient underwent primary cesarean delivery at 39 weeks for breech presentation with APGARs 7 and 9, at 1 and 5 min respectively. Neonate weighed 3,380 g. Both mother and neonate were discharged home in stable condition without adverse events.

## Case 2

### Symptoms/signs

29-year-old G3P1102 was exposed to Parvovirus at 13 weeks’ gestation and experienced fever, headache, nausea, and emesis. Past medical history was significant for preterm premature rupture of membranes (PPROM) and preterm labor (PTL) at 32 weeks in a prior pregnancy.

#### Diagnosis/treatment

Parvovirus titers were noted to be elevated to IgM 5.6/IgG 2.4 at 15 weeks, MCA-PSV was >2 MoM without hydrops at 17 weeks (patient declined PUBS due to procedure-related risks), and 1.83 MoM at 18 weeks (patient was amenable to IVIG). Patient was transfused IVIG 1  g/kg daily × 2 doses (total 120 g) at 18 weeks. Parvovirus titers were noted to be IgM 2.14/IgG 5.62 at 18 weeks (pre-IVIG), and post IVIG dropped to IgM 0.2/IgG 6.8 at 30 weeks. Maternal serum viral counts were 3.8 log copies/mL at 18 weeks, 5.11 log copies/mL at 20 weeks, and 3.07 log copies/mL at 30 weeks.

Incidentally on anatomy survey, fetus was found to have a hypoplastic aorta for which amniocentesis was done, which confirmed positive PCR for Parvovirus B19 DNA.

#### Follow-up

**Figure 1:**
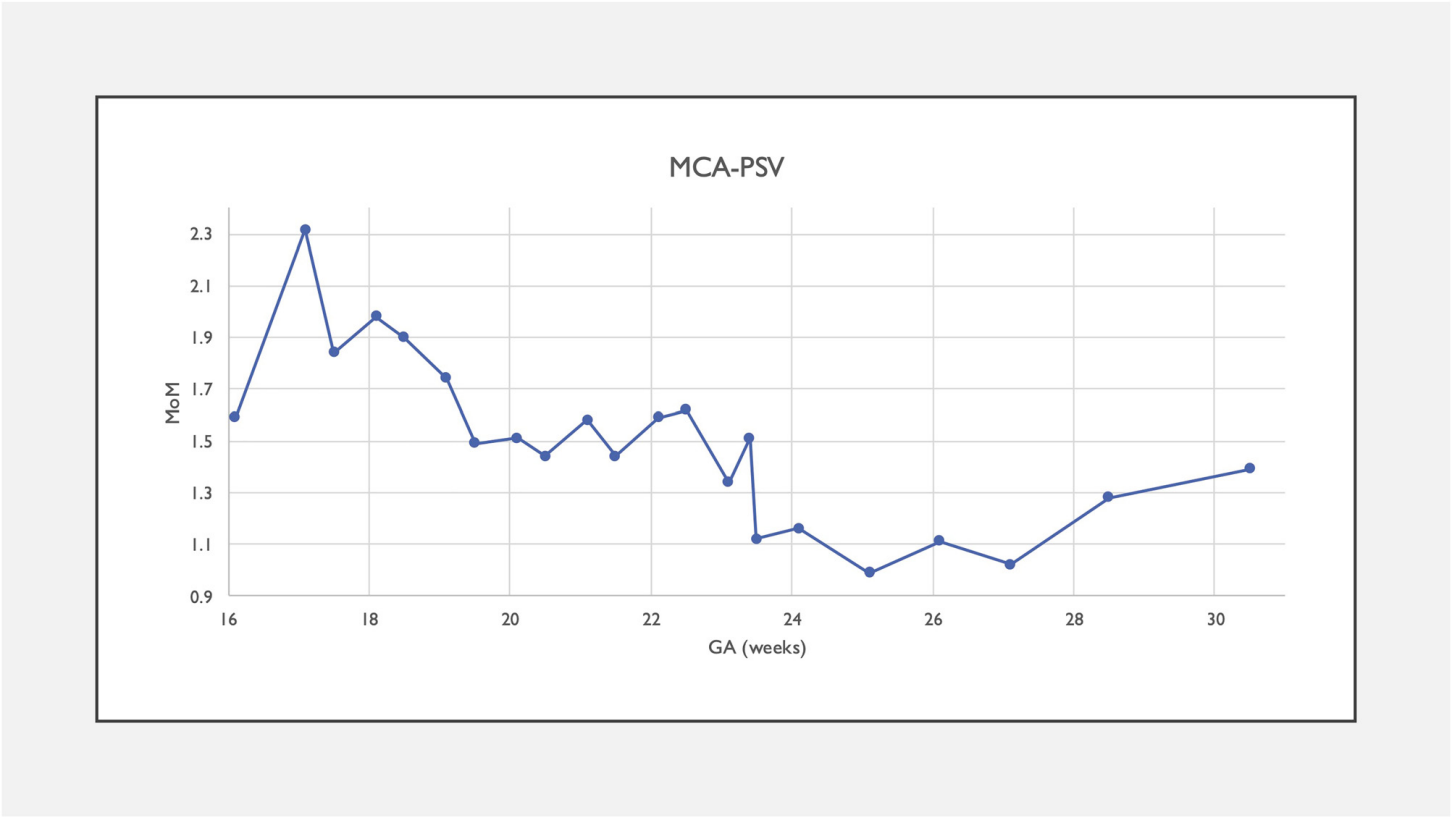
MCA-PSV trend throughout pregnancy pre- and post-IVIG administration (case 2).

We monitored reticulocyte counts for bone marrow response alongside MCA-PSV for 12 weeks post-IVIG. Reticulocyte count remained high post-IVIG at 2.8 (8 days post-IVIG) with decreased MCA-PSV at 1.74 MoM (10 days post-IVIG) and normalization at 1.49 MoM (14 days post-IVIG, see Figure 1).

The patient presented in early labor at 38 weeks and had an uncomplicated vaginal delivery with APGARs 9 and 9, at 1 and 5 min respectively. Neonate weighed 2,920 g. Both mother and neonate had uncomplicated courses without adverse events.

## Discussion

The cases presented show successful reduction in Parvovirus B19 viral load and prevention/recovery of bone marrow suppression after IVIG treatment of acute infection with Parvovirus B19. Unlike in prior studies [4], our patient’s viral load in case 2 initially increased after IVIG treatment despite MCA-PSV normalization but consistent with prior studies had a slow clearance of viral load months after the initial diagnosis [6]. In a Swedish study of -5 patients, B19 DNA PCR (peripheral viremia) persisted after development of IgG and symptom resolution with delayed viral clearance after 106 days in 4 out of -5 patients [6]. Thus, viral load levels and clearance rates are variable and are not accurate predictors of fetal status. In a prospective study of 20 mother-fetus pairs who had IUT, maternal and fetal viral loads did not predict the severity of B19-induced fetal anemia; therefore, ultrasound was recommended as the modality for identifying fetal anemia [7]. As better reflections of maternal and fetal status, maternal reticulocyte counts and fetal MCA-PSV values should guide intervention. We recommend treatment with IVIG based on low reticulocyte counts and/or high MCA-PSV values to prevent fetal hydrops after consultation with hematology to determine the ideal dosage frequency, which was done for this case series.

Risk-stratifying which patient would benefit from high-dose IVIG prior to onset of fetal hydrops may be helpful for the treatment of acute Parvovirus B19 in pregnancy. Per SMFM guidelines, reference ranges for MCA-PSV are established from 18 to 40 weeks [8]; in our patient at low risk for hematopoietic suppression (case 2), there is utility in risk-benefit discussion with the patient to offer IVIG less than 18 weeks’ gestation as an alternative to PUBS/IUT with risk of fetal loss at 1–2 % and higher risk with earlier gestations [9].

Thus, IVIG may be useful for Parvovirus B19 treatment in patients with erythropoietic suppression, in patients who decline PUBS/IUT with elevated MCA-PSV, and in patients who have difficult access to the placental cord insertion site, unsuccessful attempt at PUBS, or relative contraindications (i.e. significant abdominal surgery, high BMI). However, given limited information in the third trimester, there is a need for prospective studies to assess the efficacy of IVIG in the third trimester.

## Take-home messages

1. Treatment with IVIG based on low reticulocyte counts and/or high MCA-PSV values may be considered to prevent maternal/fetal complications.
2. IVIG may be useful for Parvovirus B19 treatment in patients with erythropoietic suppression, in patients who decline PUBS/IUT with elevated MCA-PSV, and in patients who have difficult access to the placental cord insertion site, unsuccessful attempt at PUBS, or relative contraindications (i.e. significant abdominal surgery, high BMI).
